# Severe Plasmodium falciparum Malaria Complicated by Post-artemisinin Delayed Hemolysis in a Non-immune Pediatric Returning Traveler

**DOI:** 10.7759/cureus.103322

**Published:** 2026-02-09

**Authors:** Sara Galadari, Madeeha Kalsekar, Asif Iqbal, Zarife Daoud, Maida Balila, Omar Nyanga

**Affiliations:** 1 Medicine, Mohammed Bin Rashid University of Medicine and Health Sciences, Dubai, ARE; 2 Internal Medicine/Infectious Disease, Mediclinic City Hospital, Dubai, ARE; 3 Pediatric Infectious Diseases, Mediclinic Middle East, Dubai, ARE; 4 Infectious Disease, Mediclinic Welcare Hospital/Mediclinic City Hospital, Dubai, ARE; 5 Laboratory Technical Services, Mediclinic City Hospital, Dubai, ARE

**Keywords:** artemisinin-based combination therapy, artesunate, case report, hemolytic anemia, malaria, plasmodium falciparum, post-artemisinin delayed hemolysis

## Abstract

Severe *Plasmodium falciparum* malaria remains a major cause of morbidity in children, particularly among non-immune travelers. Intravenous artesunate is the treatment of choice for severe malaria due to its rapid parasite clearance and improved safety profile. However, post-artemisinin delayed hemolysis (PADH) is increasingly recognized as a delayed adverse effect, typically occurring seven to 30 days after treatment. Recognition of PADH is crucial to avoid misdiagnosis, guide monitoring, and ensure timely intervention.

A previously healthy nine-year-old boy presented with severe *P. falciparum* malaria following travel to Tanzania. He developed multiorgan dysfunction, including thrombocytopenia, acute kidney injury, hepatic dysfunction, and hypoxemic pneumonia, and was treated in the pediatric intensive care unit with intravenous artesunate followed by oral artemether-lumefantrine. After clinical improvement and parasite clearance, he was discharged. Ten days after treatment initiation, he re-presented with pallor, jaundice, and dark urine. Laboratory evaluation demonstrated hemoglobin decline, indirect hyperbilirubinemia, elevated lactate dehydrogenase (LDH), reticulocytosis, low haptoglobin, and absence of parasitemia - confirming PADH. He required admission and transfusion of packed red blood cells, with subsequent full hematological recovery.

This case highlights PADH as an important delayed complication of artesunate therapy in non-immune pediatric travelers. Early recognition, structured post-treatment monitoring, and clear differentiation from malaria recrudescence are essential to guide management. This case reinforces the need for follow-up hemolysis testing seven to 30 days after treatment, particularly in patients with high initial parasitemia.

This case report describes a child with severe falciparum malaria successfully treated with intravenous artesunate who subsequently developed PADH. It illustrates the biphasic course of artemisinin therapy - initial parasite clearance followed by delayed hemolysis - underscoring the need for post-discharge monitoring.

## Introduction

Severe *Plasmodium falciparum* malaria remains a life-threatening condition, particularly in young children and non-immune travelers returning from endemic regions [1]. Artemisinin derivatives, particularly intravenous artesunate, have transformed severe malaria management through rapid parasite clearance and reduced mortality [2], compared with quinine [3]. Despite this benefit, a distinct delayed hemolytic syndrome - post-artemisinin delayed hemolysis (PADH) - has been increasingly reported [4]. PADH typically arises seven to 30 days after treatment and is attributed to splenic “pitting,” whereby once-infected erythrocytes are returned to circulation with shortened life spans. PADH may result in clinically significant anemia that can require transfusion, and can be mistaken for treatment failure or malaria recrudescence. Although PADH is well-described in African children treated for severe malaria, reports among non-immune pediatric travelers remain rare. This case illustrates a classic presentation of PADH following artesunate therapy and emphasizes the need for structured post-treatment monitoring.

## Case presentation

A previously healthy nine-year-old boy presented with a four-day history of high-grade fever, chills, vomiting, diarrhea, and malaise after returning from Tanzania, where he had been visiting family. On admission, he was febrile, tachycardic, lethargic, dehydrated, and visibly jaundiced. Examination revealed mild hepatosplenomegaly, clear sensorium, and no focal neurological deficits.

**Figure 1:**
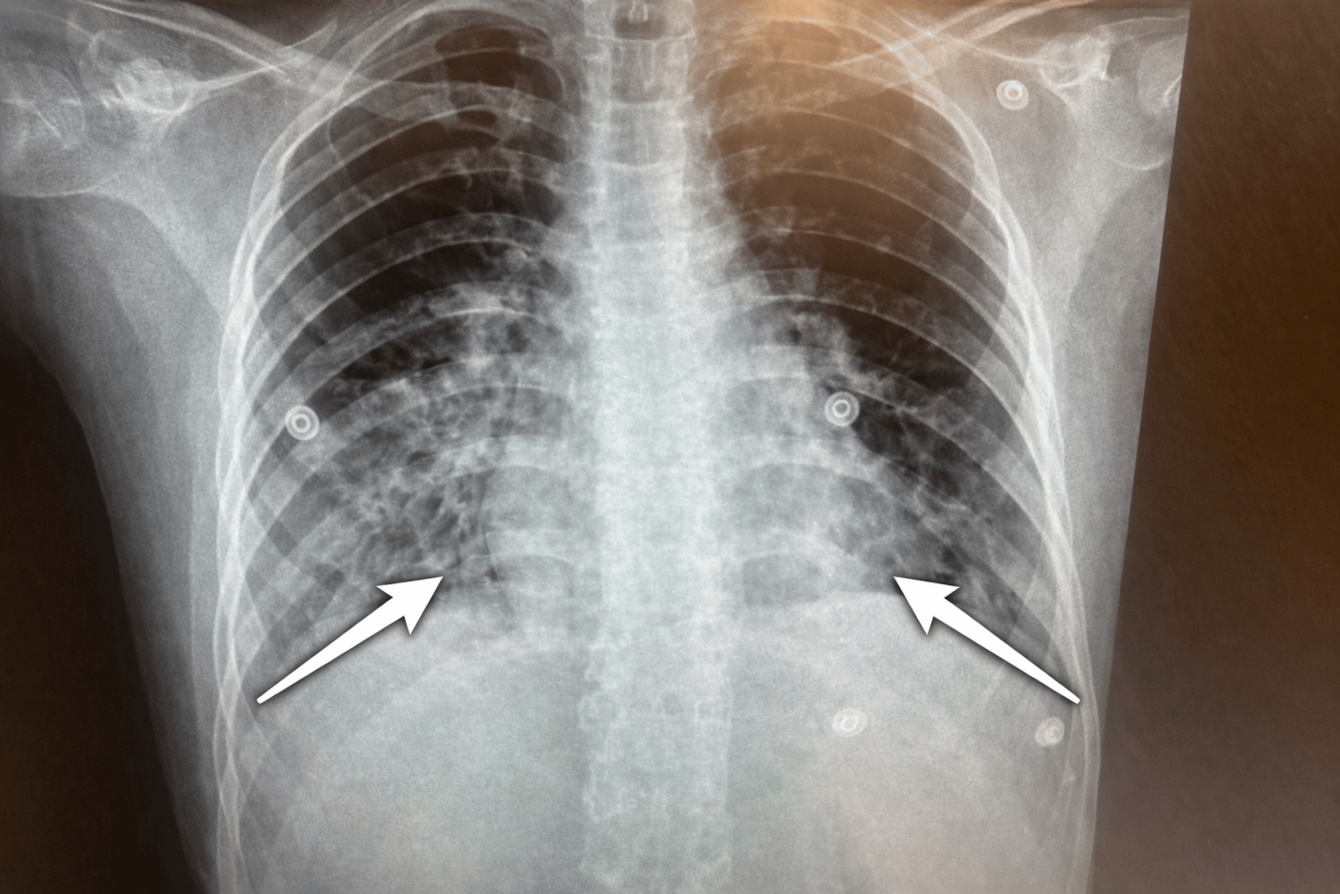
Chest radiograph demonstrating bilateral basal infiltrates (white arrows)

Peripheral smear confirmed *P. falciparum* infection with a parasite index of 6.1% (moderate parasitemia >5%) [5]. Laboratory results, summarized in Table 1, showed thrombocytopenia (16,000/µL), hemoglobin 10.5 g/dL, hyperbilirubinemia, acute kidney injury (creatinine 1.24 mg/dL; baseline 0.59 mg/dL), and elevated inflammatory markers. Chest radiograph demonstrated bilateral basal infiltrates (Figure 1). Respiratory viral PCR was positive for respiratory syncytial virus (RSV).

**Table 1:** Laboratory findings during initial admission and readmission

Laboratory Parameter	Initial Admission	Readmission	Follow-up	Reference Range	Units
Hemoglobin	10.5	7.4	11.2	11.5-15.5	g/dL
Platelet count	16,000	343,000	294,200	150,000-450,000	/µL
Serum creatinine	1.24	-	0.59	0.3-0.7	mg/dL
Lactate dehydrogenase (LDH)	665	1037	275	192–321	U/L
Total bilirubin	52.9	26.1	12.6	0.8-9.4	µmol/L
Direct bilirubin	38.7	15.6	4.5	0.0-5.0	µmol/L
Indirect bilirubin	14.2	10.5	4.7	2.8-22.9	µmol/L
Haptoglobin	-	0.47	3.33	0.50-2.29	g/L
Reticulocyte count	-	12.0	1.97	0.42-2.23	%
Absolute reticulocyte count	-	564.7	83.71	42-70	×10⁹/L
Parasite index (*Plasmodium falciparum*)	6.1	0.0	0.0	0	%
Direct antiglobulin test (DAT)	-	Negative	Negative	Negative	Negative

He was admitted to the pediatric intensive care unit (PICU) and treated with intravenous artesunate, intravenous ceftriaxone, fluid resuscitation with electrolyte correction, and oxygen via high-flow nasal cannula. By day 3, parasitemia cleared. He received two apheresis platelet units, with improvement to 225,000/µL. Renal function normalized (creatinine 0.54 mg/dL). After completing four days of intravenous artesunate, he was transitioned to oral artemether-lumefantrine for a three-day course and discharged clinically stable with hemoglobin 10.8 g/dL.

Ten days after treatment initiation, the patient returned with fatigue, jaundice, and dark (“cola-colored”) urine but no fever. Laboratory evaluation, also summarized in Table 1, revealed hemoglobin 7.4 g/dL, elevated lactate dehydrogenase (LDH) (1037 U/L), indirect hyperbilirubinemia, low haptoglobin, and reticulocytosis (12%). Repeat malaria smears were negative, and the direct antiglobulin test (DAT/Coombs) was negative.

The findings were consistent with PADH. He was re-admitted and received one unit of packed red cells, with hydration and folate supplementation. His hemoglobin stabilized and rose to 8.2 g/dL prior to discharge. Follow-up testing two weeks later showed complete hematologic recovery (hemoglobin 15.9 g/dL).

## Discussion

This case illustrates the dual impact of artemisinin therapy - rapid parasite clearance followed by delayed hemolytic anemia. PADH has been increasingly recognized as a delayed complication of artemisinin therapy. For example, a retrospective cohort in Spain [6] reported delayed hemolysis in four of 21 severe malaria patients treated with IV artesunate, appearing nine to 14 days after treatment and requiring supportive transfusions.

Although most reports involve intravenous artesunate, recent studies confirm that delayed hemolysis can also occur after oral artemisinin-based combination therapy (ACT) [7]. A large prospective observational study of travelers with uncomplicated *P. falciparum* malaria treated with three-day oral ACT [8] found that about 37.4% of patients exhibited laboratory-defined hemolysis on day 14, although most cases were subclinical. More severe cases have been reported, including fatal outcomes in individuals who declined transfusion [9]. 

Mechanistically, the leading hypothesis is splenic “pitting” of once-infected erythrocytes [10]. Artesunate (and ACT) kill the parasite within erythrocytes, but remnants are removed in the spleen; the erythrocytes then re-enter circulation with a reduced lifespan (often seven to 21 days). Higher initial parasitemia increases the pool of once-infected RBCs, amplifying the risk and severity of PADH. Immune-mediated hemolysis has also been reported, with occasional DAT positivity [6], and rare cases treated with corticosteroids.

Our case aligns with this growing body of literature and illustrates that PADH is not exclusive to intravenous artesunate and may follow oral ACT [4]. However, in our patient, PADH followed intravenous artesunate given for complicated malaria, highlighting the risk of higher morbidity in the pediatric population. The non-immune status of this child is particularly relevant, as non-immune travelers often present with higher parasite densities than semi-immune individuals in endemic regions. This larger initial mass of infected red cells translates into a greater burden of once-infected erythrocytes after treatment, predisposing to more clinically significant PADH.

## Conclusions

PADH is an uncommon but clinically significant complication of artemisinin therapy that is increasingly recognized following both intravenous artesunate and oral artemisinin-based combination therapy. This case adds to the growing evidence that hemolysis can emerge after treatment for severe malaria and may require hospitalization. Clinicians should maintain a high index of suspicion for this complication, particularly in high-risk groups such as children, pregnant women, and individuals with pre-existing anemia.

We recommend a practical post-treatment follow-up protocol in which complete blood count and hemolysis markers, including lactate dehydrogenase and bilirubin, are repeated at seven to 10 days and again at three to four weeks following artesunate therapy, particularly in patients with an initial parasitemia greater than 4%. Patients and their families should be counseled regarding the warning signs of hemolysis, such as fatigue, pallor, jaundice, and dark urine, and instructed to seek immediate medical attention should these occur. Children are especially vulnerable to anemia-related complications and therefore require close post-treatment monitoring.
